# Editorial

**Published:** 1915-06

**Authors:** 


### EDITORIAL

The Business Office of the *Journal-Record* is 23 West Alabama Street.

The Editorial Office is 708 Empire Life Building.

Address all Business Communications to *Journal-Record of Medicine*, 23 West Alabama Street.

Make remittances payable to *The Journal-Record of Medicine.*

On matters pertaining to the Editorial and Original Communications, address **Edgar G.**

Reprints of Original Articles will be furnished at cost price. **Requests for the same** should always be made in THE MANUSCRIPT.

Richard R. Daly, M. D.

We will present, postpaid, on request to each contributor of an original **article,** twenty (20) marked copies of *The Journal-Record of Medicine* containing **such** article.

---

### ASSOCIATION MEETING.

The Chattahoochee Valley Medical and Surgiacl Association will hold its fifteenth Annual Session at Opelika, Alabama, on July 3 and 14, and a general invitation is extended to all physicians within convenient distance to attend. An excellent program has been arranged and it is printed elsewhere in the Journal.

This Association serves a special purpose in a successful manner. The local Societies of the various Counties and Districts are limited somewhat in their effects by the fact that the members become familiar with each other and are able to pressage what each man is likely to say. The State Societies and the larger groupings such as the Southern Medical are on the other hand, a bit diffused so far as the social part is concerned and men do not get well enough acquainted with each other except in limited instances, to be able to value the scientific contributions correctly. The special field of the Chattahoochee Valley Association gathers in men of a common interest who are not otherwise likely to get acquainted and at the same time does not establish a convention that is unwieldy. The members learn to know each other well and to be able to get what each has to offer for the advancement of science without ever feeling a familiarity that is sometimes deadening.

The meetings are well attended and papers are of pronounced value. We hope to publish many of them later on.

Dr. W. J. Love, the present Secretary of the Association, may well be said to be its Father, for it was due to his foresight that it was organized and carried through its early days. The coming meeting is to be held at his home, and this alone would insure to all who know him, a delightful time. Dr. Langley, as President, and the other officers have combined to make this Session a memorable one well worthy of every doctor's attendance.

---

### SURGICAL RESPONSIBILITY.

In the preparation for his first operation the mind of the novitiate is filled with eagerness and pleasant anticipations. The various anatomical problems have been foreseen and settled and the other matters of technic have been rehearsed. The anticipation ons one of success and thoughts of risk are minimized by the recollection of previous instruction as well as by the lack of experience of loss. In all probability, too, there is to be present an associate to whom one may turn for help if needed.

The first incision is made with a steadiness that is forced so that throbbing pulses will not mar the work or cloud the eye and as the operation proceeds to a sucecssful issue, thrills of pleasure and satisfaction fill the mind and the man for the first time in his life, perhaps, feels that he is made. The enthusiasm of a new experience is grafted upon that of youth and strength and it is increased by the aspirations for greater work and extending fame which this the first actual step has signalized.

The responsibility has been measured by the relation between the work done and the instructions the young surgeon has been receiving. He has done just what his elders have told him and all is well. With the new hopes come confidence and daring and aggressiveness. Let us remove the offending material quickly and neatly and have at once the relief surgery will bring instead of waiting weary weeks and suffering through conservative delay. The thing is there and under the splendid scientific conditions evolved during the last twenty-five years, we can do anything we dare do. The responsibility is one of judgment and nerve—a knowledge of the condition and the skilled determination to relieve it.

And then the years pass and leave their burdens and their treasures to ripen the mind and clarify the judgment of the surgeon. Daring becomes a thing of the past. It has no place in surgery. No man has ever a right to risk anything involving any but himself. Enthusiasm has changed to a deep appreciation of the wonderful opportunities and results of curative measures, and responsibility has extended so as to embrace not only the technic of the immediate work but the remote results with all their undefined risks which experience alone can bring to a man's equipment. Courage grows and this means doing things that one is somewhat afraid to do.

Even the simplest of operations now means that it has been considered in the light of all the successes and all the failures the years have brought and that nothing is too small or insignificant not to have thoughtful attention. Nothing can be slighted; every preparation must be accurate and complete and the accidental contingencies must be foreseen. The dangers face one and they are overcome by courage and the habit of doing things right, but they are not set aside and forgotten. The surgeon of greatest experience takes the fewest chances; indeed, he takes none at all outside those that are inevitable in human frailty and idiosyncracy. He knows what he is about and what he will do every instant. He realizes the responsibility he is carrying and it shows in the dignity of his bearing as well as in the care of his work. He has come to the point where he hesitates and takes stock before beginning things and holds back the urgent enthusiasm of his younger associates because matters of life and death as they are placed in his hands with every section he makes are more to him than the mechanical process of the operating room.

The man who has had ten years of experience in general or special surgical work and who has in that time gained the confidence of his patients and fellows is habitually barred from taking chances. His sense of responsibility has ripened and grown as from day to day and week to week, he has taken the lives of his friends and their dear ones in his hands until every case demands all he is capable of giving to it and nothing is done lightly or unadvisedly and nothing is omitted. Not only is there careful thought, but the surgical habit of years of growing caution and exactitude is present to insure correct procedure.

Once undertaken the work must go through and nothing outside can make a demand upon him. Expediency has no place near him. His whole interest is concentrated in the work in hand whether it be complicated or simple because of the habit and of the responsibility his life has brought to him.

It is absurd and ridiculous to think that it could be otherwise and when a surgeon has been accused of violating the fundamental principles of antisepsis and surgery itself, it behooves us to bear these things in mind and to decide whether it would be possible.

Responsibility in finance and its corelated matters is of great importance and much if not most of our happiness and contentment in life depend upon its exercise. Men are held by it with strong and durable bonds. Responsibility in surgery comprehends life and death and the man who has had enough experience to realize this binds himself ever closer to meet its demands. He can not be driven to evade them.

